# Utilizing Molecular Descriptor Importance to Enhance Endpoint Predictions

**DOI:** 10.3390/toxics13050383

**Published:** 2025-05-09

**Authors:** Benjamin Bajželj, Marjana Novič, Viktor Drgan

**Affiliations:** 1 Laboratory for Cheminformatics, Theory Department, National Institute of Chemistry, Hajdrihova 19, 1001 Ljubljana, Slovenia; benjamin.bajzelj@ki.si (B.B.); marjana.novic@ki.si (M.N.); 2 Biotechnical Faculty, University of Ljubljana, Jamnikarjeva 101, 1000 Ljubljana, Slovenia

**Keywords:** quantitative structure–activity relationship, molecular descriptors, molecular descriptor importance, enzyme inhibition, hepatotoxicity

## Abstract

Quantitative structure–activity relationship (QSAR) models are essential for predicting endpoints that are otherwise challenging to estimate using other in silico approaches. Developing interpretable models for endpoint prediction is valuable as interpretable models may provide valuable insights into the relationship between molecular structure and observed biological or toxicological properties of compounds. In this study, we introduce a novel modification of counter-propagation artificial neural networks that aims to identify key molecular features responsible for classifying molecules into specific endpoint classes. The novel approach presented in this work dynamically adjusts molecular descriptor importance during model training, allowing different molecular descriptor importance values for structurally different molecules, which increases its adaptability to diverse sets of compounds. We applied the method to enzyme inhibition and hepatotoxicity classification datasets. Our findings show that the proposed approach improves the classification of molecules, reduces the number of neurons excited by molecules from different endpoint classes, and increases the number of acceptable models. The proposed approach may be useful in compound toxicity prediction and drug design studies.

## 1. Introduction

The ability to accurately predict endpoints, such as toxicity and biological activity, is essential for drug discovery and risk assessment of chemicals. Quantitative structure–activity relationship (QSAR) models use molecular descriptors for the representation of molecules to establish relationships between their molecular structure and observed properties. Identification of relevant molecular descriptors is important for improving the accuracy and reliability of the models. Understanding the importance of molecular descriptors in endpoint predictions enables better model interpretability and understanding of how changes in molecular structure may affect the endpoint. This may be important in evaluating the properties of existing compounds and in the design of new ones, where finding a balance between the desired property values and the risks (i.e., toxicity) associated with the use of chemicals is necessary. The interpretation of models may be model-based, as in linear or post hoc models, which is usually the case for machine learning models [1,2]. Linear models are simple to interpret as the target property is linearly dependent on predictors. Interpretation of artificial neural network (ANN) models is considerably more difficult; thus, the name “black box” models is frequently used. Neural network models have been successfully applied in many different fields, including protein design [3,4]; molecular simulations [5,6]; prediction of molecular properties [7,8,9]; and analysis of histological images [10,11]. Only few methods have been developed for interpreting machine learning models [12]. Some of these methods are Anchors [13], LIME [14], MCR [15], and Shapley value-based methods [16,17].

QSAR models are important for modeling and explaining complex physicochemical and biological processes [1,18,19,20]. This was also recognized by the Organisation for Economic Co-operation and Development (OECD), which addresses the fifth principle for the validation of QSAR models in its guidelines, indicating “a mechanistic interpretation, if possible” [21]. The guidelines provide some examples of how interpretation could be performed. Mechanistic interpretation could provide information on how a particular molecular substructure affects the studied property (mechanism of action) or provide physicochemical interpretation of the selected descriptors and their effect on the known or proposed mechanism. While it is accepted that not all models can be mechanistically interpreted, the development of new methods for explanation of (QSAR) models may lead towards acceptance of the fact that in some way all models can be interpreted and that useful knowledge can be extracted from them [1].

The article by Fjodorova and Novič [22] showed the possibilities of using counter-propagation artificial neural network (CPANN) models for mechanistic interpretations. They were able to relate selected molecular descriptors to known structural alerts for carcinogenicity that are mechanistically or statistically linked with cancer induction. For example, functional group count descriptor nRNNOx (number of N-nitroso groups (aliphatic)) and atom-centered fragment descriptor N-078 (-Ar-N=X/X-N=X) could be linked with known structural alert “alkyl and aryl–N-nitroso groups” for carcinogenic compounds. DNA adducts can be formed after metabolic activation of N-nitroso groups [23]. An example of using relative importance in a CPANN model is described in the work of Kuzmanovski et al. [24], where the adjustment of descriptor importance is performed prior to model training. Adjustments of importance values were carried out using genetic algorithm optimization in that case. The results showed that simpler, efficient models using relative importance could be found when compared to more complex models where descriptors have equal importance as in the standard CPANN algorithm.

In this paper, we propose a new algorithm that takes into account relative importance of molecular descriptors used in counter-propagation artificial neural network models. The CPANN method has been shown to be efficient in the modeling of various endpoints. The CPANN-v2 algorithm proposed in our previous publication [25] was used in this study as the initial algorithm that was modified so that the importance of the descriptors could be adjusted using descriptor and endpoint values during the training of the network. In the new algorithm, the adjustment of the relative importance on a neuron resembles model weight correction in the standard CPANN training so that, in a similar way, the adjustment of relative importance on the neuron decreases with increased topological distance from the central neuron. With this improved version of the CPANN-v2 algorithm, a larger number of acceptable models constructed under the same training conditions are commonly found when compared to the original CPANN-v2 algorithm. The efficiency of the new algorithm is shown and compared with previous studies on examples of enzyme inhibition datasets and hepatotoxicity datasets for the classification of chemicals.

## 2. Materials and Methods

### 2.1. Datasets

The performance of the algorithm using the relative importance of descriptors was evaluated on classification datasets available in published scientific articles. The classification datasets for the inhibition of eight enzymes and a curated hepatotoxicity dataset, based on LiverTox database [26] with pre-computed molecular descriptors, were taken from the study of Drgan et al. [25]. The original datasets of inhibitors for these enzymes were published by Sutherland et al. [27]. Sutherland’s datasets include inhibitors for angiotensin-converting enzyme (ACE), acetylcholinesterase (ACHE), benzodiazepine receptor (BZR), cyclooxygenase-2 (COX2), dihydrofolate reductase (DHFR), glycogen phosphorylase b (GPB), thermolysin (THER), and thrombin (THR). Models for these enzymes were built using pre-computed QuBiLS-MIDAS molecular descriptors that can be obtained from the original dataset. The dataset curated from the LiverTox database contained an unbalanced number of compounds in the hepatotoxic and non-hepatotoxic classes that were described using 49 molecular descriptors. This dataset is labeled “hepatotoxicity (49 desc.)”. A dataset with a balanced number of compounds in the hepatotoxic and non-hepatotoxic classes was obtained from the study by Bajželj et al. [28], where the dataset had been compiled from multiple sources. In this case, the molecules in the dataset were presented using 98 molecular descriptors, and the dataset is labeled “hepatotoxicity (98 desc.)”.

### 2.2. Counter-Propagation Artificial Neural Networks

The algorithm used in this study is a modification of the standard CPANN algorithm, which was described in detail by Zupan et al. [29,30]. The standard CPANN architecture consists of two layers of neurons stacked one above the other. The first (upper) layer of neurons, also called Kohonen layer, is used to group objects (molecules) according to their similarity, and the second layer of neurons, called the output layer or the Grossberg layer, is used to predict target properties. Neuron weights in the Kohonen layer can be represented as a 3D matrix of *Nx* × *Ny* neurons with *Ndesc* weights on each neurons. *Nx* and *Ny* denote the number of neurons in the *x*- and *y*-direction, respectively, and *Ndesc* denotes the number of molecular descriptors used to represent each object (molecule). The output layer has the same number of neurons as the input layer. The number of weights on each neuron in the output layer equals the number of target properties (*Ntar*) that the model predicts. In this study, one biological activity is predicted by all models, so *Ntar* = 1.

In the Kohonen layer, unsupervised learning is used during training, which requires descriptor values for training, and no information about the target property is needed. Euclidean distance is commonly used to find the central neuron (or the winning neuron) in the Kohonen layer. The central neuron is the most similar neuron to the input object (molecule) and has the smallest Euclidean distance to the input object. After the central neurons are identified, the weights on these neurons are corrected so that they become more similar to the input object. The largest correction of the model weights is made on the central neuron, and the corrections decrease with increasing topological distance from the central neuron according to neighborhood function (*h*). The triangular neighborhood function was used in this study, which means that the extent of corrections linearly decreases with increased topological distance from the central neuron.

In the second layer (the output layer), supervised learning is used, and target property values of objects are needed for the correction of the weights in this layer. The weights in the output layer are corrected so that they become more similar to the target property. The position of the central neuron in the Kohonen layer is projected on the output layer, and corrections of weights are made using the same equation as in the Kohonen layer, except now target property values are used instead of descriptor values.

The corrections of neuron weights w(*t*, *i*, *j*, *k*) for variable *k* (descriptor or target property) on the neuron at position (*i*, *j*) using object variable *k* (*o*(*k*)) at iteration *t* are made using Equation (1). In Equation (1), *η*(*t*) represents the learning coefficient at iteration *t*, which usually linearly decreases from its predefined maximal value to the minimal value during training.

*w*(*t*, *i*, *j*, *k*) = *w*(*t* − 1, *i*, *j*, *k*) + *m*(*t*, *i*, *j*, *k*) ∙ *η*(*t*) ∙ *h*(*i*, *j*, *t*) ∙ (*o*(*k*) − *w*(*t* − 1, *i*, *j*, *k*)) (1)

The standard CPANN algorithm does not have the term *m*(*t*, *i*, *j*, *k*) in Equation (1) and can be considered as 1 during the entire training process. This term is used in the CPANN version 2 algorithm presented in the previous study [25], which is calculated according to Equation (2).

*m*(*t*, *i*, *j*, *k*) = [1 − (1 − *p*(*t*)) ∙ ABS[scaled(*o*(*k*)) − scaled(*w*(*i*, *j*, *k*))]] ∙ [1 − (1 − *p*(*t*)) ∙ ABS[scaled(*o*(target)) − scaled(*w*(*i*, *j*, target))]] (2)

In Equation (2), *p*(*t*) linearly decreases during training from 1 to 0. ABS indicates the calculation of the absolute value in brackets. The terms scaled(*o*(*k*)), scaled(*w*(*i*, *j*, *k*)), and w(*i*, *j*, target) represent range-scaled values of object variable *k*, its corresponding range-scaled neuron weight, and the range-scaled target value of the object *o*, respectively. When all objects from the training set are used once during training, one epoch of training is finished. Predictions from the model are obtained so that first, the central neuron is found in the Kohonene layer, and then, its position is projected on the output layer. The prediction of the target property is made by reading the value of the neuron weight in the output layer.

### 2.3. Algorithm for the Estimation of Descriptor Importance

The modified CPANN version 2 algorithm, described in [25], was used for the training of neural network models. Weighted Euclidean distance was used in this study to determine central neurons, considering the importance of descriptors. In the calculation of Euclidean distance, descriptor importance can be considered a factor that defines how much weight a particular descriptor has. Weighted Euclidean distance (*EDw*) can be written in the form of Equation (3).

(3) $$EDw=\sqrt{\sum_{k=1}^{Ndesc}impn\left(k\right)\cdot {\left(o\left(k\right)-w\left(k\right)\right)}^{2}}$$

In Equation (3), *impn*(*k*) represents the normalized importance values for descriptor *k*, *Ndesc* is number of all descriptors, *o*(*k*) is the normalized value of descriptor *k* of object *o*, and *w*(*k*) is the neural network model weight for descriptor *k*. Initially, all the descriptors can be considered equally important. The correction of descriptor importance is made in each training iteration step, *t*, on all neurons where the neural network model weights are also corrected. The descriptor importance is corrected according to Equations (4)–(6). The amount of correction of descriptor importance on a neuron at position x = *i* and y = *j* is calculated using Equation (4). In Equation (4), *scalingf* represents the manually defined scaling factor, *nit* is number of all iterations (equal to the number of objects multiplied by the number of training epochs), *p*(*t*) is a linearly decreasing function from 1 to 0, *h*(*i*,*j*,*t*) is neighborhood function (triangular decreasing function in this study), and the ABS(x) function represents the absolute value of the argument x. New values for descriptor importance were calculated according to the rules presented in Equations (5) and (6). The importance of descriptors on a neuron at position (*x* = *i*, *y* = *j*) are increased if prediction at the neuron is correct for the current object; otherwise, they are decreased (Equation (5)). The correctness of the prediction can be determined using the current model output weight value and the object endpoint value. For example, in a classification problem, the prediction can be considered correct if the absolute difference between the predicted response and the actual object endpoint value differ for less than 0.5. In order to have comparable importance values across all neurons in the model, which is important for the determination of the central neuron using *EDw*, all importance values are normalized using Equation (6) so that at every neuron the sum of all descriptor importance values equals to 1. The correction of the importance value for descriptor *k* can be performed immediately after the model weight for descriptor *k* has been corrected, but the normalization of the descriptor importance is made after all impf values on the neuron have been calculated.

(4) $$delta(t,i,j,k)=\left(\frac{scalingf}{nit}\right)\cdot \left(1-p\left(t\right)\right)\cdot h\left(i,j,t\right)\cdot \frac{1}{\frac{1}{2}+2\cdot {\left(o\left(k\right)-w\left(k,i,j\right)\right)}^{2}}$$

(5) $$impf\left(t,i,j,k\right)=\left\{\begin{array}{c} impn\left(t-1,i,j,k\right)+\mathrm{delta}\left(t,i,j,k\right),\;correct\;prediction\\ impn\left(t-1,i,j,k\right)-\mathrm{delta}\left(t,i,j,k\right),\;incorrect\;prediction \end{array}\right.$$

(6) $$impn\left(t,i,j,k\right)={impf\left(t,i,j,k\right)}^{2}/\sum_{k=1}^{Ndesc}{impf\left(t,i,j,k\right)}^{2}$$

### 2.4. Calculations

The modified CPANN-v2 algorithm using descriptor importance, described in Section 2.3, was used to build classification models for enzyme inhibition (eight enzymes) and two hepatotoxicity datasets. The same descriptors and division of compounds into training, test, and validation sets were used as in the articles where the datasets were obtained. Descriptor values in all sets were normalized using means and standard deviations, which were calculated using descriptor values in the training set. The enzyme inhibition datasets had a small number of descriptors and were used to initially evaluate the performance of the proposed algorithm. Multiple models were developed for the enzyme inhibition dataset, using all descriptors and different training parameters, such as the number of neurons and minimal and maximal learning rate. Genetic algorithm (GA) optimization of multiple models was performed on hepatotoxicity datasets, and comparison of the results between different algorithms was carried out. GA optimizations were used to select the optimal set of descriptors in models and to optimize training parameters (minimal and maximal learning rate). Different random seeds were used to randomize initial network weights and the order of training set compounds in each epoch using different random seeds for a random number generator. In this way, multiple models were obtained for the same scaling factor (*scalingf*) value. The same training parameters that were used for these models were also applied to models with other scaling factor (*scalingf*) values. The results obtained using the modified CPANN-v2 algorithm were compared with the result obtained using the unmodified CPANN-v2 algorithm.

The number of acceptable models obtained when using different learning algorithms was one of the measures used to evaluate the proposed modification of the CPANN-v2 algorithm. The same criteria for the acceptability of models were used as in article [25]. Models for enzyme inhibition datasets (no GA optimizations) were accepted if sensitivity and specificity in the training and external validation sets were at least 0.7. Models for hepatotoxicity datasets were accepted if the average sensitivity and specificity in the training, test, and external validation sets were at least 0.7 when considering 100 models built with different permutations of objects using the same training parameters to build neural network models. The models that were accepted are referred to as “acceptable models” within this article.

## 3. Results and Discussion

Counter-propagation artificial neural network (CPANN) models for the classification of chemicals were built using the CPANN version 2 algorithm (CPANN-v2), which is described in article [25]. In this study, the algorithm was further extended so that the newly modified training algorithm allowed adjustments of descriptor relative importance during the training process. The new algorithm allowed different importance values for the same descriptor on different neurons of the model. With such a modification, it is made possible for very structurally different compounds to show high importance values for different descriptors while being in the same endpoint class. That is in line with the fact that different molecular fragments may be responsible for the activity of compounds. A number of models were built using classification datasets to verify the performance of the proposed algorithm. The analysis of the results was focused on the descriptor importance and its relation to the endpoint, accuracy of the models, and how these results are affected by the scaling factor (*scalingf*, see Equation (4)) that guides the magnitude of descriptor importance adjustments.

### 3.1. The Influence of Scaling Factor on the Number of Acceptable Models

**Figure 1:**
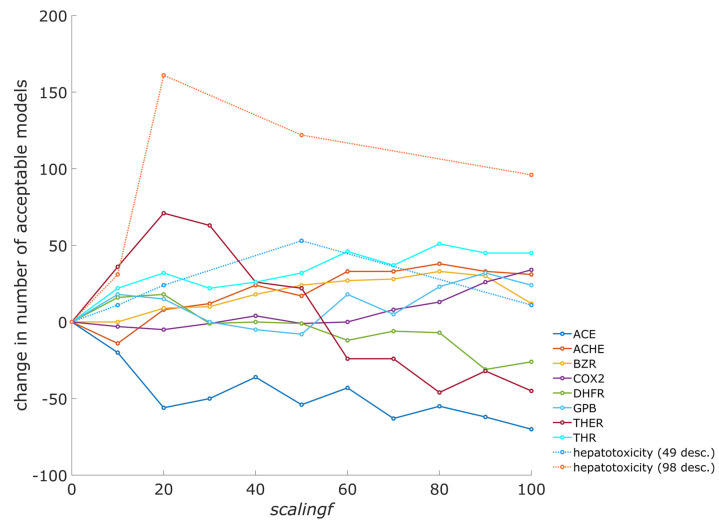
Effect of scaling factor (*scalingf*) on the number of acceptable models. Dataset abbreviations: ACE—angiotensin-converting enzyme; ACHE—acetylcholinesterase; BZR—benzodiazepine receptor; COX2—cyclooxygenase-2; DHFR—dihydrofolate reductase; GPB—glycogen phosphorylase b; THER—thermolysin; THR—thrombin, hepatotoxicity (49 desc.)—unbalanced liver toxicity dataset with 49 molecular descriptors; hepatotoxicity (98 desc.)—balanced liver toxicity dataset. Only the number of acceptable models belonging to the same dataset may be compared, as the training conditions and number of models built varied between the datasets. Within the same dataset, calculations were repeated using the same training conditions, except the value of the *scalingf* parameter, to obtain the results shown in the figure. The number of different calculations performed at each *scalingf* parameter value was 360 for ACE and ACHE, 420 for BZR, 660 for COX2, 720 for DHFR, and 300 for GPB, THER, and THR. For both hepatotoxicity datasets, 1440 optimization runs were performed, and the best five models were tested using an external validation set. The graph was created using the data available in the Supplementary Materials (in zip files).

**Figure 2:**
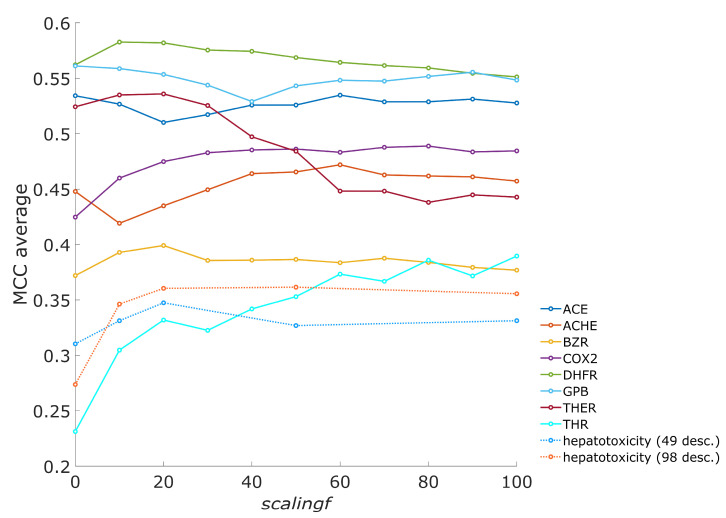
Averages of Matthews correlation coefficient (MCC) for validation sets over all models.The same models were used as for Figure 1.

The scaling factor (*scalingf*) is important for the algorithm as it governs the magnitude of corrections of descriptor importance values on neurons in the neural network learning process. When the differences in the descriptor importance are large, the descriptors with large importance contribute the most to the total weighted Euclidean distance, while descriptors with small importance have a negligible contribution. Considering that the central neuron for an object is the neuron with the smallest Euclidean distance to the object, a small importance value for a descriptor on one neuron, while having high value on other neurons, can importantly affect central neuron selection. Different descriptor importance values on neurons can lead to significant changes in the grouping of the compounds and, consequently, in model performance. A number of models were built to assess how importance values affect performance of the models. Different scaling factors, *scalingf* (see Equation (4)), were used to build models for the inhibition of eight enzymes and two hepatotoxicity datasets. For enzyme datasets, all descriptors in the datasets were used in the models (from six to nine descriptors), while for both hepatotoxicity datasets, genetic algorithm optimizations of models were performed. The effect of the scaling factor on the number of acceptable models is presented in Figure 1, where the change in the number of acceptable models is shown when compared to the results where no scaling factor was used (i.e., when an unmodified CPANN-v2 training algorithm is used). A larger number of acceptable models can be found when a modified CPANN-v2 algorithm employing descriptor importance is used, except in the case of angiotensin-converting enzyme (ACE). Calculations for each dataset were performed under the same training conditions when using a modified or unmodified algorithm, and only the value of the scaling factor (*scalingf*) was altered. Results showing prediction accuracy and acceptance of models are available in the Supplementary Materials (files: enzyme_inhibition_datasets_results.zip, hepatotoxicity_dataset_results.zip, and livertox_dataset_results.zip). The trends in Figure 1 indicate that an increase in the number of acceptable models can be observed in a limited range of scaling factor values. Figure 2 shows the average Matthews correlation coefficient (MCC) for the validation set, calculated over all models when using different scaling factor (*scalingf*) values. An increase in the average MCC is also observed for most of the datasets in a limited range of scaling factor values.

### 3.2. Average Fraction of Neurons with Conflicts

**Figure 3:**
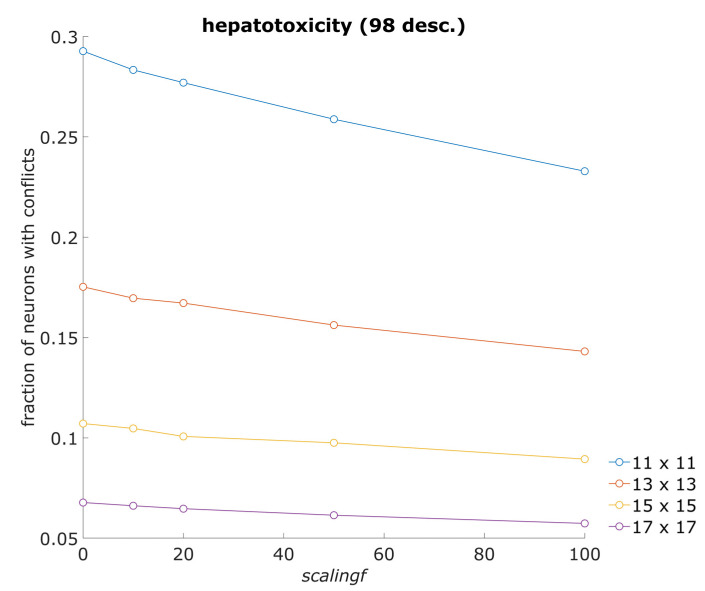
Average fraction of neurons with conflicts for hepatotoxicity (98 desc.) dataset. The average fraction of neurons with conflicts for training set objects was calculated from models using the hepatotoxicity (98 desc.) dataset. The models were built using 11 × 11, 13 × 13, 15 × 15, and 17 × 17 neurons. Figure 3 shows the average fractions of neurons with conflicts that were calculated at specific *scalingf* parameter values and network sizes.

Along with the number of acceptable models, we also examined the occurrence of neurons that are excited by objects from the training set, which belong to different endpoint classes (“neurons with conflicts”). The fraction of neurons with conflicts depends on the number of neurons in a neural network model. Therefore, average fractions of neurons with conflicts were calculated separately for models with different numbers of neurons. An example graph in Figure 3 shows results obtained using the hepatotoxicity (98 desc.) dataset. Figures showing the average fraction of neurons with conflicts for all datasets are given in the Supplementary Materials (file: Supplementary_file_S1.pdf). The average fraction of neurons with conflicts usually decreases when the scaling factor (*scalingf*) is increased, indicating that both classes of objects in the training set can be better separated when using the scaling factor (descriptor importance). The points with a value of 0 for *scalingf* correspond to the results obtained using the unmodified CPANN-v2 algorithm.

### 3.3. Descriptor Weights and Importance in Hepatotoxicity Model

**Figure 4:**
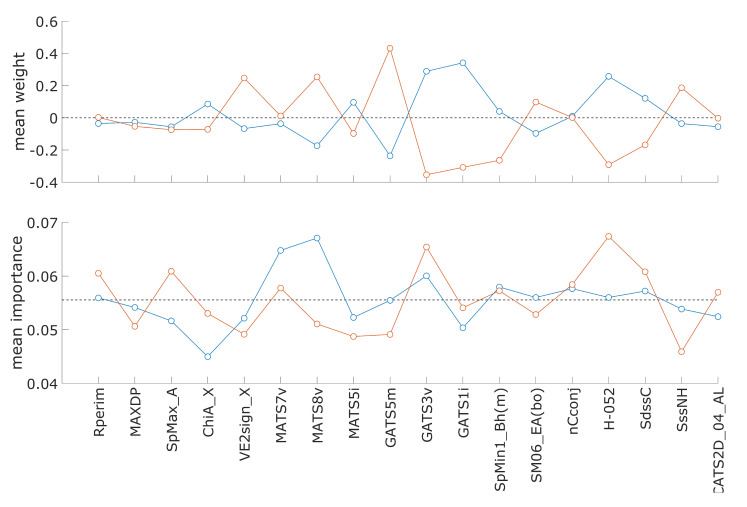
Comparison of mean weight and importance values for descriptors in the hepatotoxicity model. Blue and red markers indicate the mean values from neurons where the model predicts the non-hepatotoxic and hepatotoxic classes, respectively. The black horizontal lines in the middle of the graphs indicate zero-mean descriptor weight and an importance value of 1/18 in the upper and lower graph, respectively. A list of descriptors with their descriptions is available in Supplementary File list_of_descriptors.xlsx.

The possibility of interpretation is one of the advantages of CPANN models. The interpretation of CPANN models is commonly performed using level plots of (descriptor) model weights, which can provide clues about molecular features that are important for endpoint prediction. The modified CPANN-v2 algorithm allows different importance values of descriptors on neurons, which can be visualized in the same way as descriptor weights. The importance values are essentially also weights used in the calculation of weighted Euclidean distance, as presented in Section 2.3 (see Equation (3)). Supplementary Files Figures S1 and S2 show examples of level plots of model descriptor weights and descriptor importance, respectively. Importance values are normalized so that the sum of all importance values on each neuron equals one. Both figures were made from model data available in Supplementary File model_S1.xlsx. A list of descriptors with their descriptions is available in Supplementary File list_of_descriptors.xlsx. The model was optimized for the hepatotoxicity (98 desc.) dataset, where a larger increase in the number of acceptable models was observed when descriptor importance was used in the model (*scalingf* = 20) than in the optimizations with the hepatotoxicity (49 desc.) dataset. Using the model weights, neurons giving hepatotoxic and non-hepatotoxic prediction were identified, and mean values of the descriptor weights and descriptor importance values were calculated. The calculated means are graphically presented in Figure 4, where blue and red colors denote results for non-hepatotoxic and hepatotoxic class prediction, respectively.

Figure 4 can aid in identifying the descriptors in the model, which may have different values for compounds belonging to one of the two classes. For a better understanding of the model’s predictions, it is necessary to compare level plots, as shown in Supplementary Figures S1 and S2. A list of descriptor names with their descriptions is available in Supplementary File “list_of_descriptors.xlsx”. Descriptor mean weight values, shown in Figure 4, have larger differences for descriptors GATS5m, GATS1i, GATS3v, H-052, MATS8v, and VE2sign_X when comparing values for the hepatotoxic and non-hepatotoxic classes. Considering the mean importance values of descriptors, the largest differences are observed for descriptors MATS8v, H-052, SpMax_A, ChiA_X, and SssNH. The descriptor H-052 (“H attached to C0(sp3) with 1X attached to next C”) shows large differences in mean weight and importance values, emphasizing its importance for the prediction of the endpoint. From the level plots, it can be observed that high importance values for descriptor H-052 are on neurons resulting in positive hepatotoxic prediction.

## 4. Conclusions

The proposed algorithm for the estimation of descriptor importance using a counter-propagation artificial neural network algorithm allows the use of different importance values for the same descriptor among neurons in the artificial neural network model. The descriptor importance values are estimated during the learning process of the artificial neural network and influence the selection of central neurons and, therefore, the grouping of compounds. The flexibility in the individual descriptor importance values contributes to better discrimination between objects that would otherwise excite the same neuron or belong to the same group of objects if descriptor importance values were the same for all neurons. The algorithm was tested using enzyme inhibition datasets and hepatotoxicity datasets to develop classification models for screening and toxicity assessment of compounds. The results indicate that under the same training conditions, a larger number of acceptable models can be found with the new method than with the original algorithm that treats all descriptors as equally important. The proposed method may not always give better results. It was observed that when increasing the *scalingf* parameter, which regulates the number of corrections made to descriptor importance during model training, after reaching a certain value of the *scalingf* parameter, the number of acceptable models starts decreasing. For most of the datasets used in this study, using values of *scalingf* between 20 and 50 resulted in an increased number of models.

## Data Availability

The data are contained within the article or Supplementary Materials. Interested researchers can obtain the source code of the program used to build the CPANN models from the corresponding author upon reasonable request.

## Supplementary Materials

The following supporting information can be downloaded at: https://www.mdpi.com/article/10.3390/toxics13050383/s1, Figure S1: Level plots of descriptor weights from Supplementary File model_S1.xlsx; Figure S2: Level plots of descriptor importance from Supplementary File model_S1.xlsx; list_of_descriptors.xlsx: list of descriptors with descriptions; model_S1.xlsx: file containing model weights and descriptor importance; Supplementary_file_S1.pdf: file containing s showing average fraction of neurons with conflicts for all datasets; enzyme_inhibition_datasets_results.zip: tables for inhibition datasets showing performance of models at different values of scaling factor (*scalingf*); hepatotoxicity_dataset_results.zip: tables for hepatotoxicity (98 desc.) dataset showing performance of models at different values of scaling factor (*scalingf*); livertox_dataset_results.zip: tables for hepatotoxicity (49 desc.) dataset showing performance of models at different values of scaling factor (*scalingf*).
