# Supplementary material for: Utilizing Molecular Descriptor Importance to Enhance Endpoint Predictions
Source: Toxics. 2025 May 9;13(5):383. doi: 10.3390/toxics13050383 (PMC12115611; doi:10.3390/toxics13050383)
Supplement: Supplementary file 1 [file toxics-13-00383-s001.zip › Supplementary_files/Supplementary_file_S1.pdf]

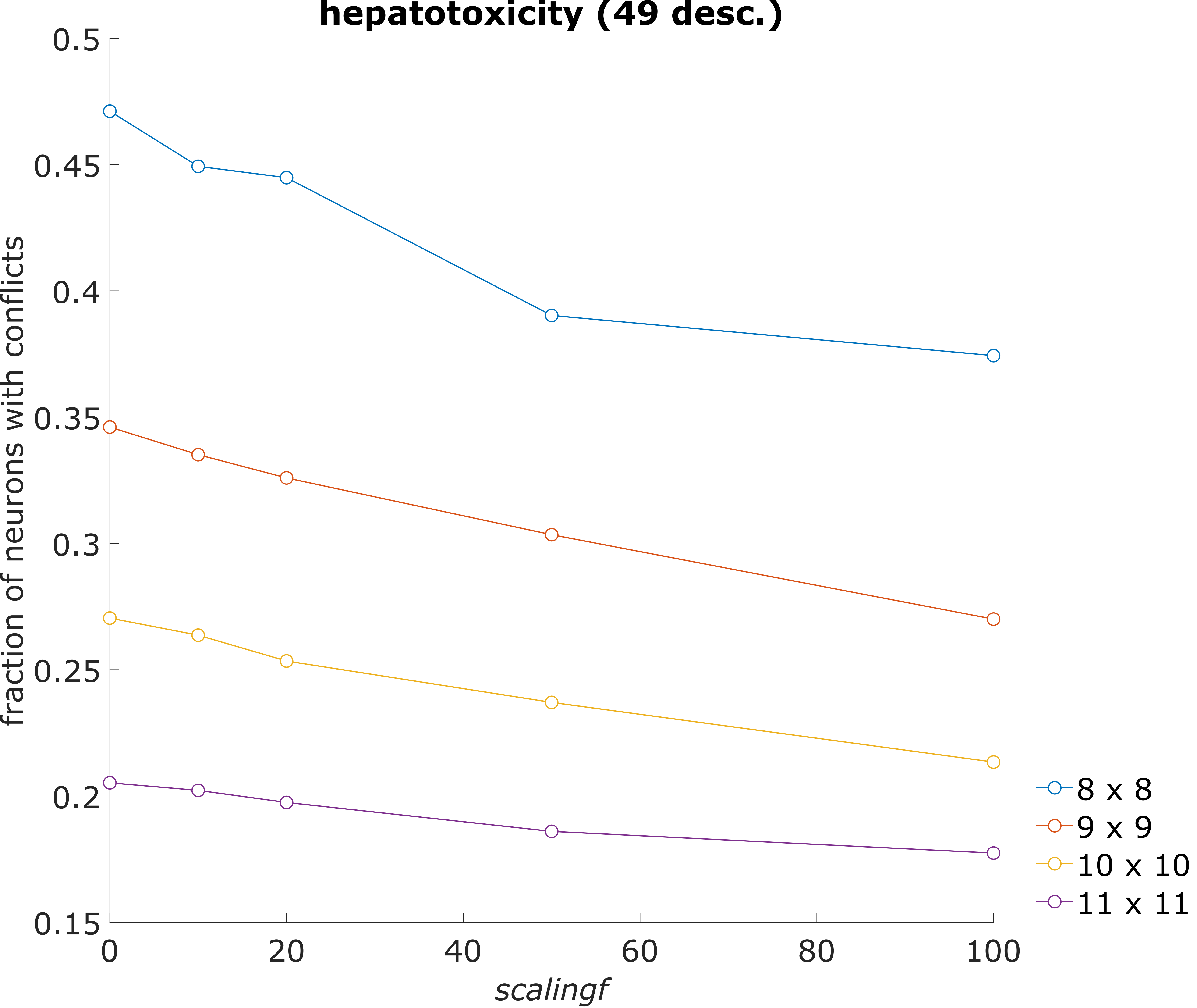
hepatotoxicity (49 desc.)
fraction of neurons with conflicts
scalingf
0.5
0.45
0.4
0.35
0.3
0.25
0.2
0.15
0
20
40
60
80
100
8 x 8
9 x 9
10 x 10
11 x 11

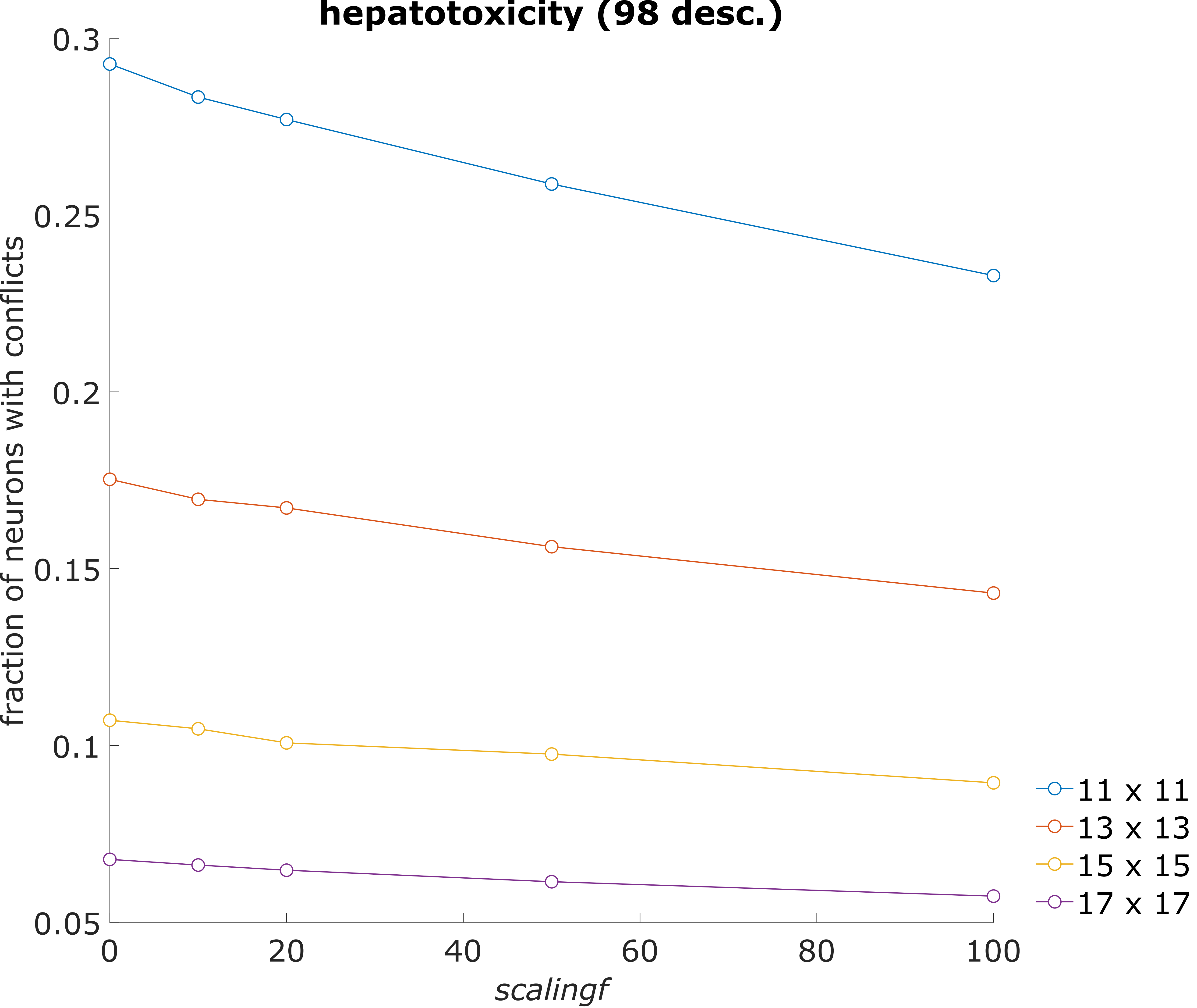

hepatotoxicity (98 desc.)
fraction of neurons with conflicts
0.3
0.25
0.2
0.15
0.1
0.05
0
20
40
60
80
100
scalingf
11 x 11
13 x 13
15 x 15
17 x 17

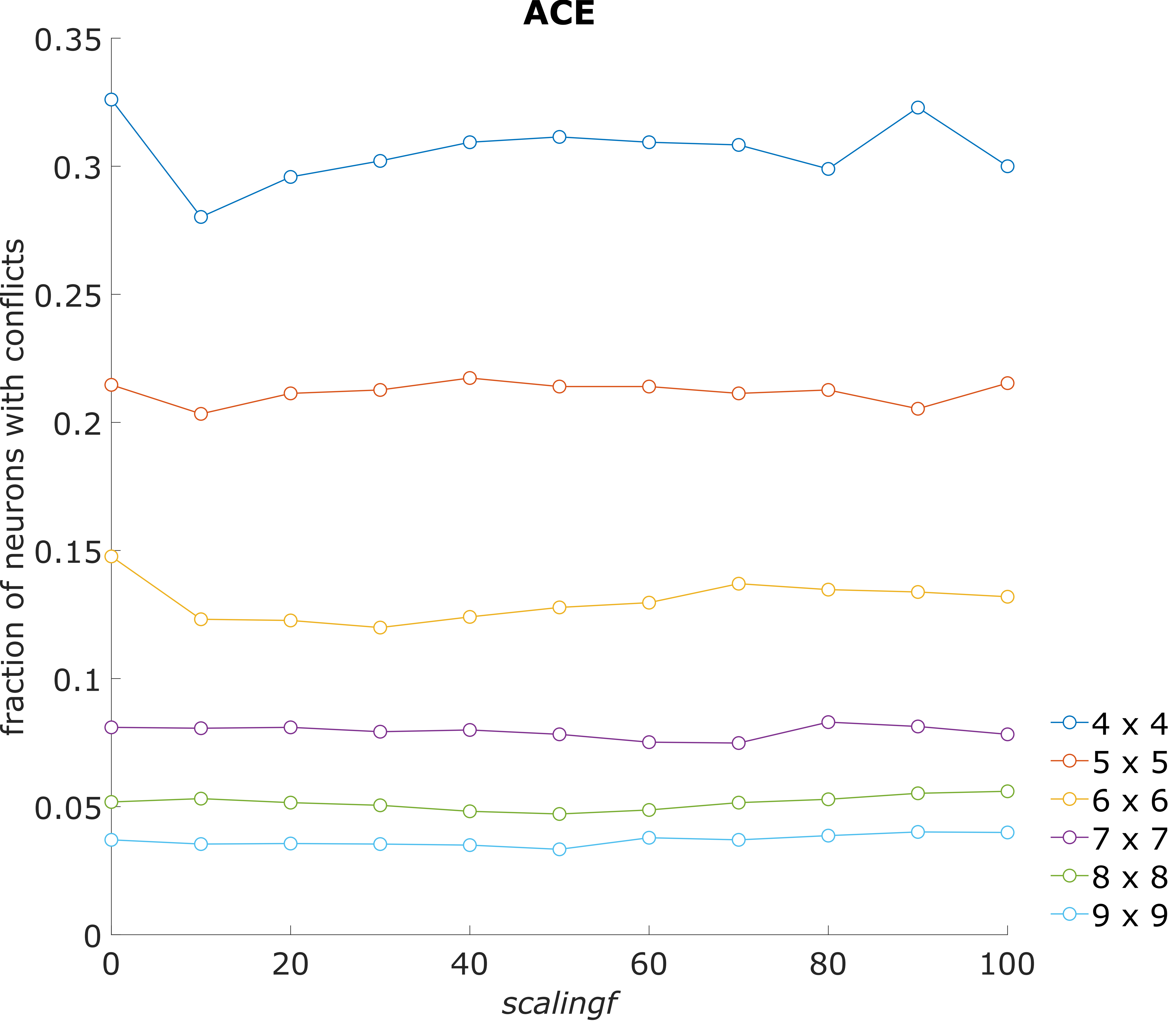

ACE
0.35
0.3
0.25
0.2
0.15
0.1
0.05
0
fraction of neurons with conflicts
0
20
40
60
80
100
scalingf
4 x 4
5 x 5
6 x 6
7 x 7
8 x 8
9 x 9

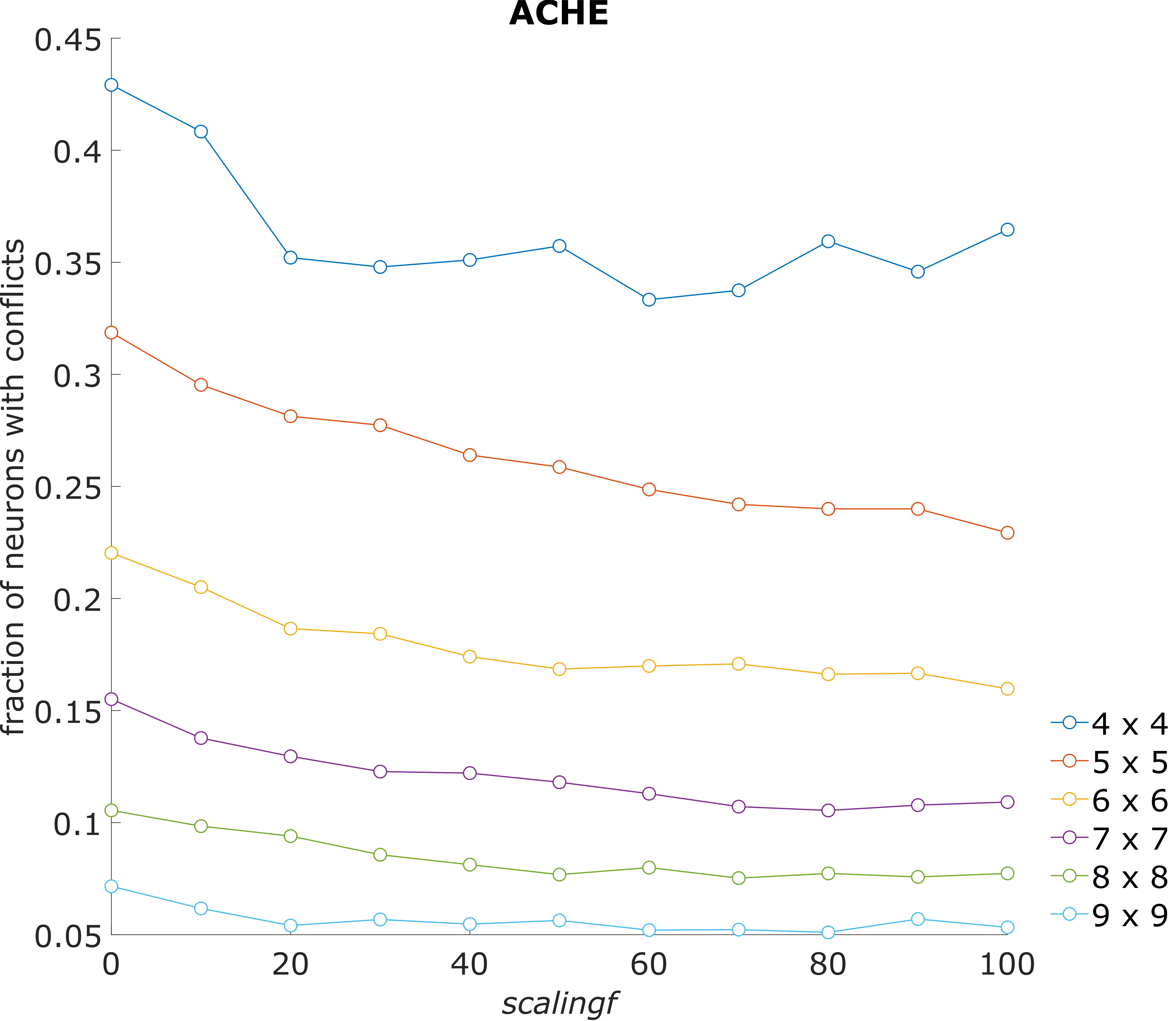
ACHE
fraction of neurons with conflicts
scalingf
0.45
0.4
0.35
0.3
0.25
0.2
0.15
0.1
0.05
0
20
40
60
80
100
4 x 4
5 x 5
6 x 6
7 x 7
8 x 8
9 x 9

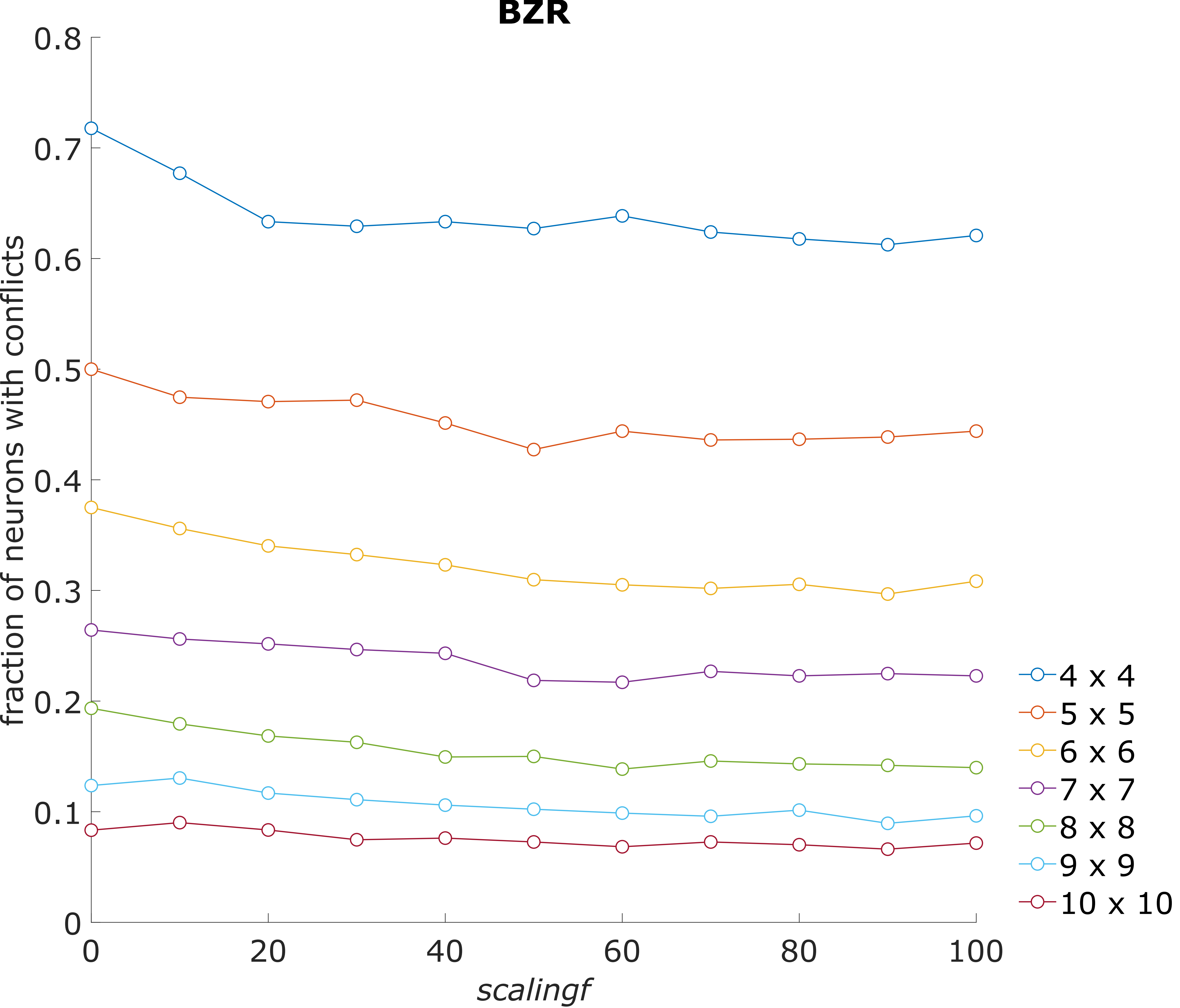
BZR
fraction of neurons with conflicts
scalingf
0
0.1
0.2
0.3
0.4
0.5
0.6
0.7
0.8
0
20
40
60
80
100
4 x 4
5 x 5
6 x 6
7 x 7
8 x 8
9 x 9
10 x 10

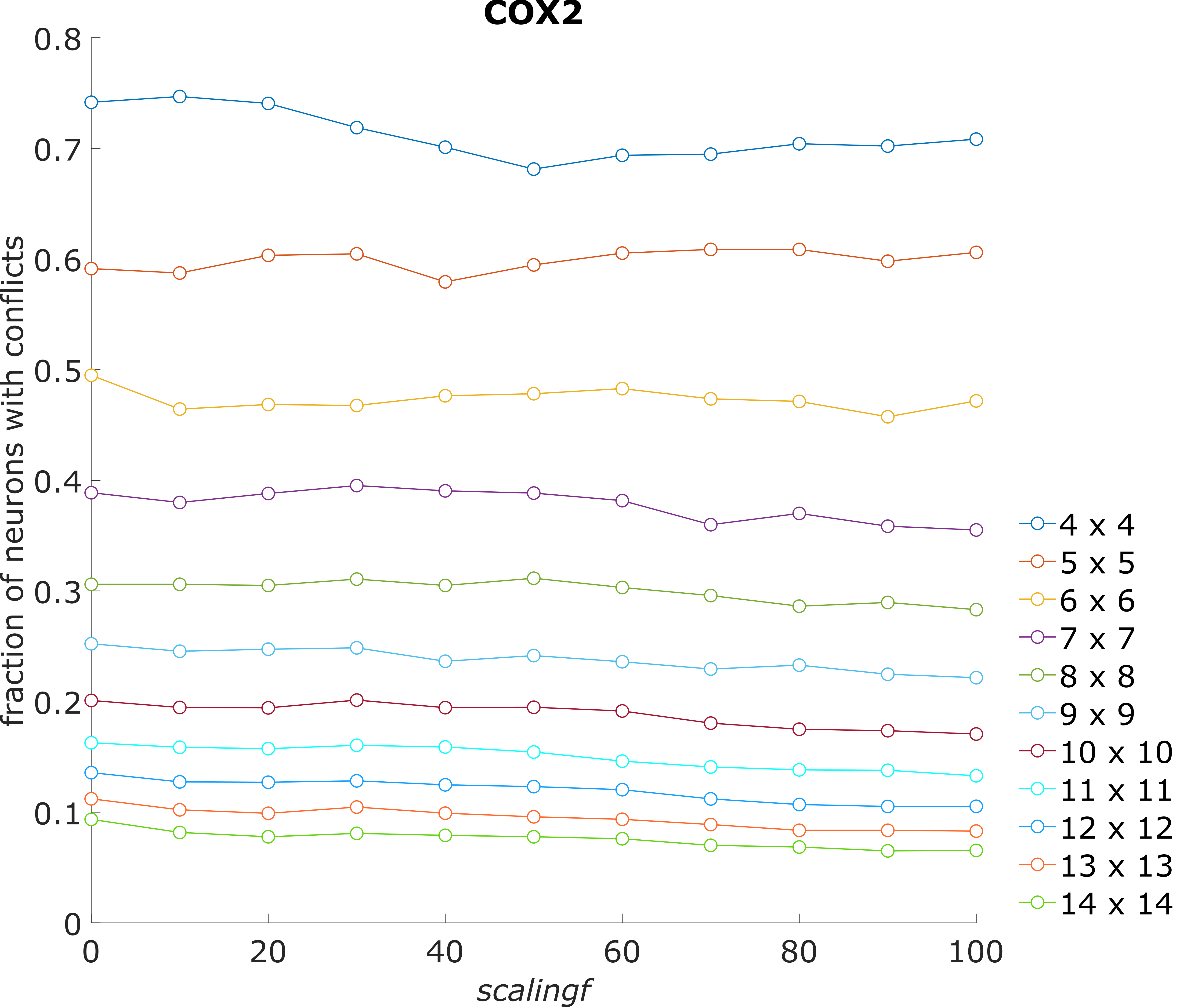
COX2
fraction of neurons with conflicts
scalingf
0
0.1
0.2
0.3
0.4
0.5
0.6
0.7
0.8
0
20
40
60
80
100
4 x 4
5 x 5
6 x 6
7 x 7
8 x 8
9 x 9
10 x 10
11 x 11
12 x 12
13 x 13
14 x 14

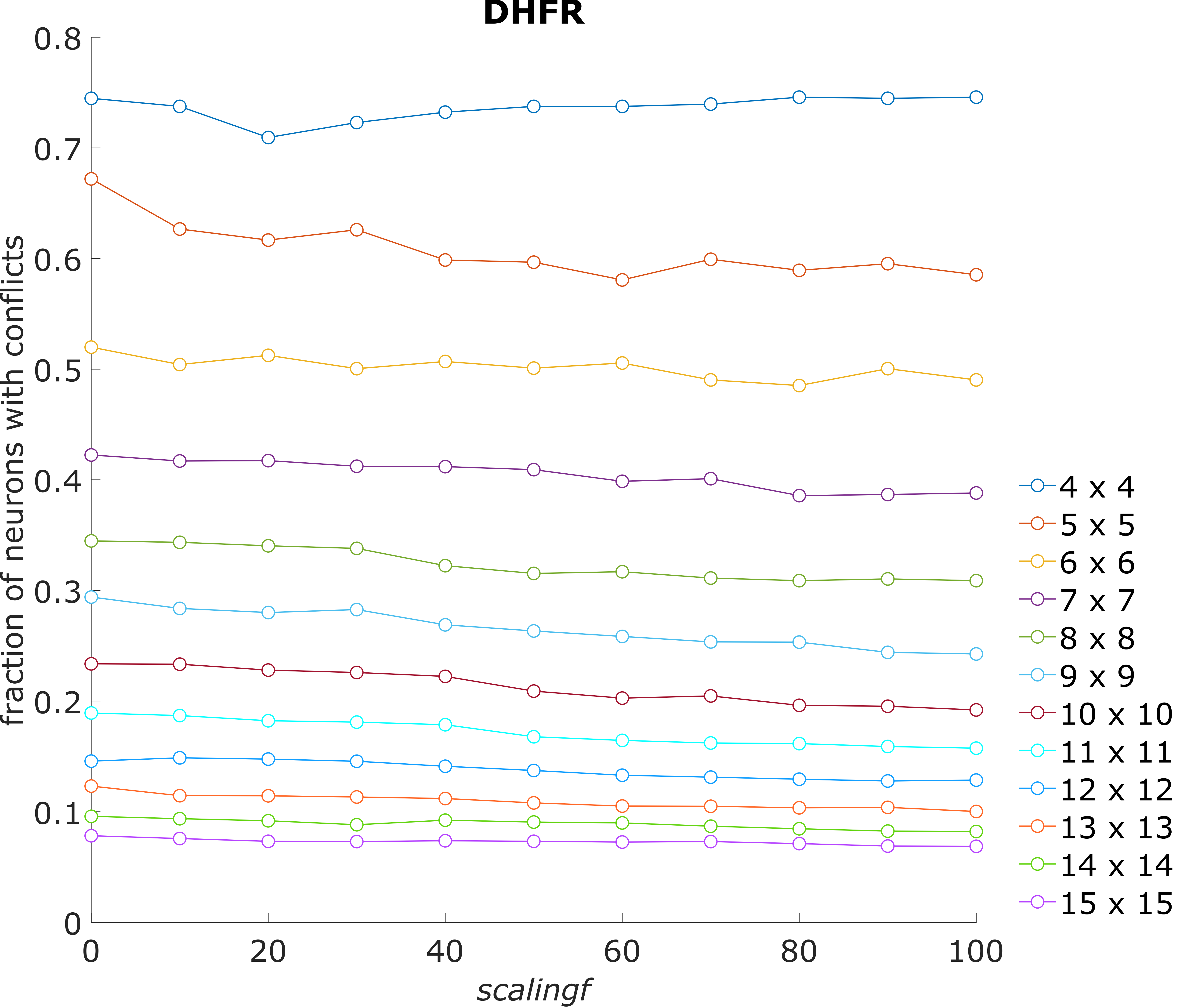
DHFR
fraction of neurons with conflicts
scalingf
0
0.1
0.2
0.3
0.4
0.5
0.6
0.7
0.8
0
20
40
60
80
100
4 x 4
5 x 5
6 x 6
7 x 7
8 x 8
9 x 9
10 x 10
11 x 11
12 x 12
13 x 13
14 x 14
15 x 15

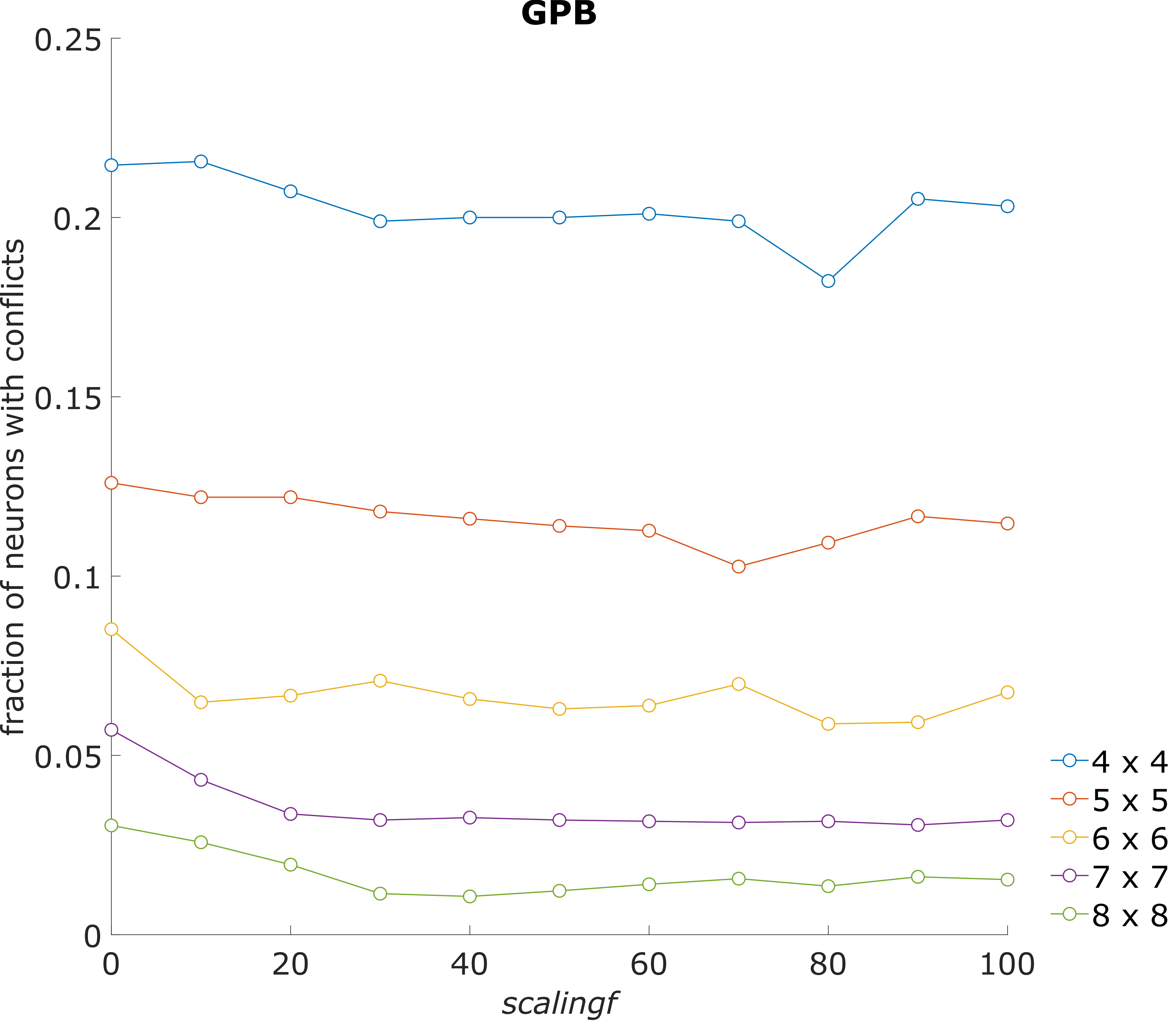
GPB
fraction of neurons with conflicts
0.25
0.2
0.15
0.1
0.05
0
0
20
40
60
80
100
scalingf
4 x 4
5 x 5
6 x 6
7 x 7
8 x 8

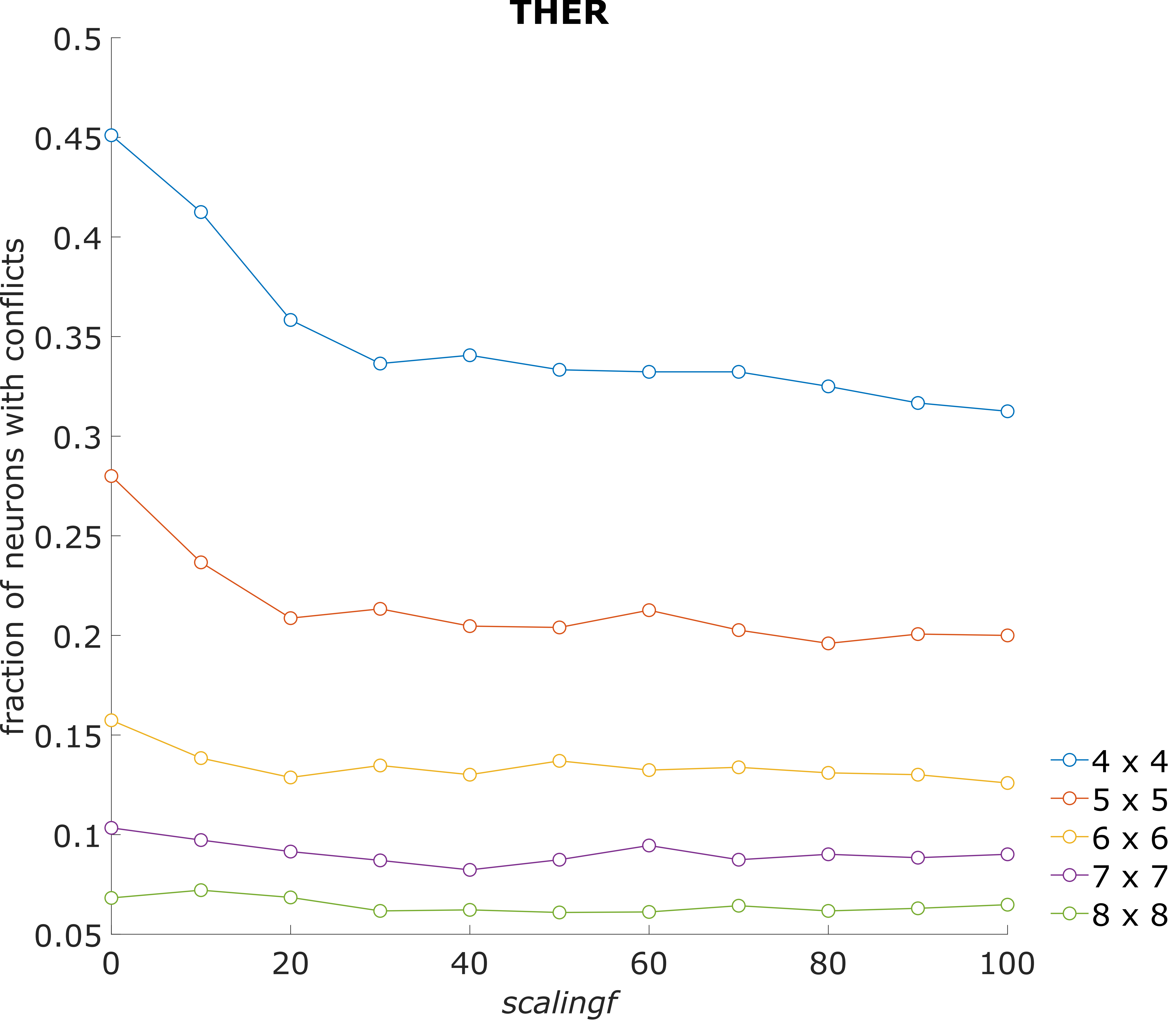
THER
fraction of neurons with conflicts
0.5
0.45
0.4
0.35
0.3
0.25
0.2
0.15
0.1
0.05
0
20
40
60
80
100
scalingf
4 x 4
5 x 5
6 x 6
7 x 7
8 x 8

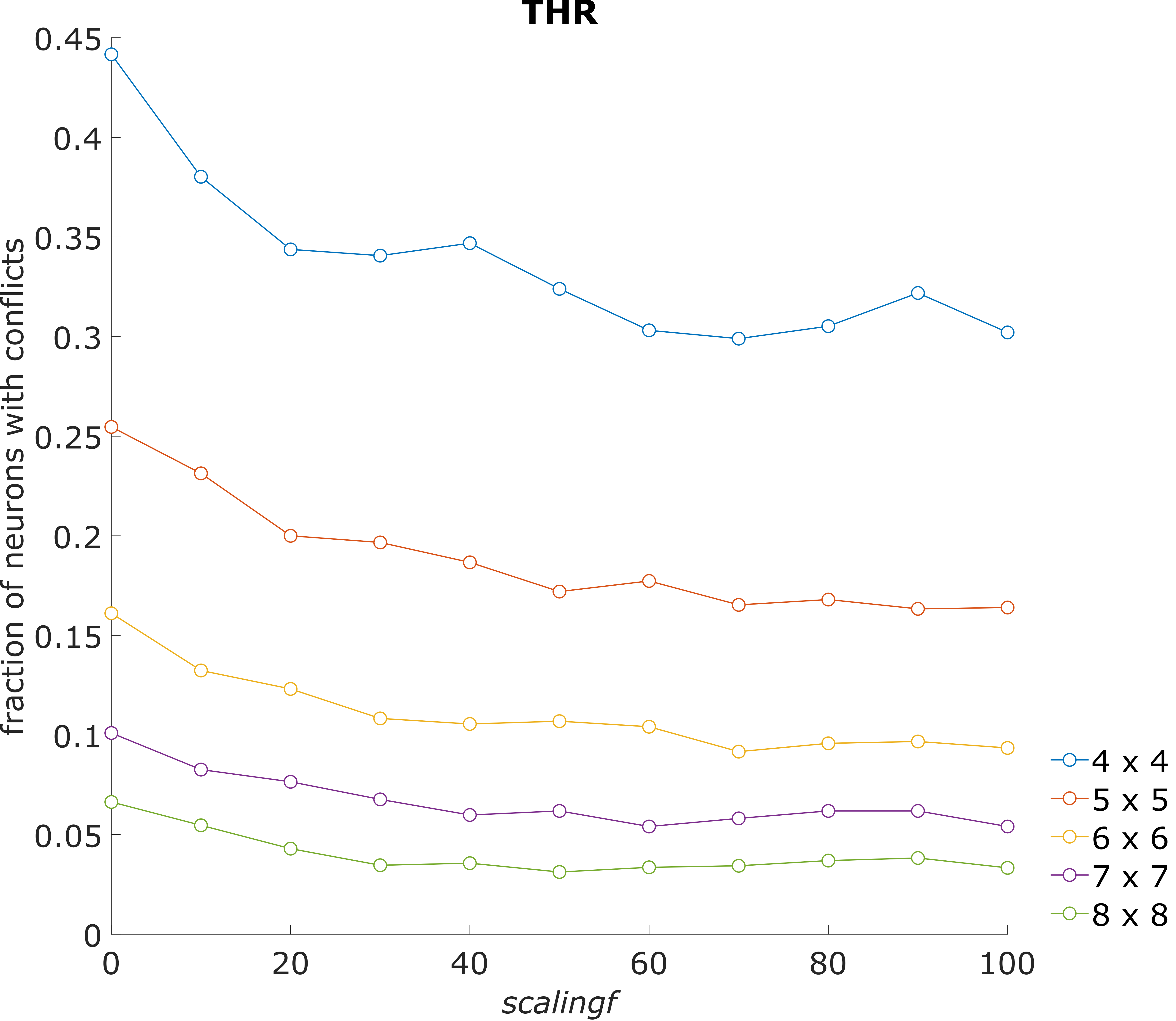
THR
fraction of neurons with conflicts
scalingf
0.45
0.4
0.35
0.3
0.25
0.2
0.15
0.1
0.05
0
0
20
40
60
80
100
4 x 4
5 x 5
6 x 6
7 x 7
8 x 8
